# Hop production in the Czech Republic and its international aspects

**DOI:** 10.1016/j.heliyon.2020.e04371

**Published:** 2020-07-11

**Authors:** Karel Šrédl, Marie Prášilová, Roman Svoboda, Lucie Severová

**Affiliations:** Faculty of Economics and Management, Czech University of Life Sciences Prague, Kamýcká 129, Prague 6, Suchdol, 165 00, Czech Republic

**Keywords:** Agricultural sciences, Economics, Chmelarstvi, druzstvo Zatec, Hops, Hops production, Hop fields, Hop yields

## Abstract

Hop-growing has had a long tradition in the Czech Republic, and it remains in the interest of the Czech economy to further develop this agricultural sector. With an almost one-tenth share of the harvest, the Czech Republic is the third largest producer of aroma hops in the world after Germany and the US, which together account for over two-thirds of the hop market. The purpose of this article is to describe the current state of the Czech and global markets in hops, and the position of the dominant company, Chmelarstvi, druzstvo Zatec, on this market. Towards the end of 2017, the area of hop fields in the Czech Republic reached 4,945 ha, which is the most in the last 7 years. Year-over-year, this represents an increase of 3.4%. The increase was caused by the current demand for quality Czech hops and the weak European harvest in 2015. Thus, the storehouses emptied, and breweries' stores all over the world, to which 80% of domestic hop production is supplied, ran out. Since then, demand and prices have increased to a level that has begun to cover the costs of hop production. This has also meant that investments in hop production have increased. With the increasing area of hop fields in the Czech Republic, hop growers have to deal with a significant problem, which is the unavailability of workers.

## 1 Introduction

Hop-growing has a great tradition in the Czech Republic and it remains in the interest of Czech farmers to further develop this sector of crop and plant production. “Exports can be associated with employment in agriculture, manufacturing and/or services, depending on the specialisation of the country regarding international trade” (Stijepic and Wagner, 2018). Hops, especially of the Saaz variety (Žatecký poloraný červeňák), are a traditional Czech export commodity. The first documented hop cultivation in Bohemia was in the 8th century CE, and hops were exported to neighbouring countries as early as the beginning of the second millennium CE.

Hop is a dicotyledonous plant of the family Cannabaceae. There are two types of hop: wild hops (ornamental plant or animal fodder) and the economically important commercial hop varieties. Hop flowers (seed cones) contain aromatic substances, resin and tannins that are important in beer-brewing (Rybáček, 1991). “Hop (Humulus lupulus L.; Cannabaceae) has a wide diversity of secondary metabolites with functional properties, such as bitter acids, essential oils and flavonoids, with health applications on lifestyle-related diseases, inflammation and antioxidants” (Sbardella et al., 2018). “One means of increasing yield and quality is the production of resistant hop lines” (Henning et al., 2017). “In the 90th, a methodology of hop breeding was innovated in the Czech Republic. Registration of a new variety Agnus in 2001 represents the result of the innovation mentioned above. Agnus variety is the first high-alpha hop in the assortment of Czech hop varieties” (Nesvadba and Krofta, 2002). “It has been necessary to prepare the quality system of Agnus identification from other Czech genotypes and characterize the germplasm of this variety by molecular methods” (Patzak, 2002).

“The bitter taste of beer is an important flavour attribute that consumers expect and enjoy to a varying degree during consumption” (Hough et al., 1982). “To impart bitterness, and hop aroma, brewers conventionally add hops (Humulus lupulus L.) to wort and boil for a duration of an hour to ninety minutes” (De Keukeleire, 2000). “This process yields the compounds agreed to be beer's major source of bitterness – iso-α-acids or isohumulones, from hop α-acids or humulones” (Hough et al., 2012).

“Both the time of hop addition and hop variety used for beer production have been suggested as factors that may impact on bitterness quality” (Hieronymus, 2012). Hieronymus adds that “Aroma hop varieties i.e. those used predominantly by brewers to impart hop aroma and flavour are also thought to contain ‘unspecific bitter substances’ which contribute positive bitterness quality when added at the onset of the boil” (Hieronymus, 2012).

“The type of hop products used and hopping regime adopted have been reported to impact on the perceived bitterness character of beer” (Oladokun et al., 2016a). “The impact of hop aroma on perceived beer bitterness has also been investigated, with findings revealing that hop aroma significantly impacts on both perceived bitterness intensity and character. Such effects are believed principally to result from taste–aroma interactions, and are potentially also impacted by trigeminal sensations elicited in the mouth by hop aroma extracts” (Oladokun et al., 2016b).

“The meaning of ‘Quality’ or ‘Character’ of bitterness remains unclear even to many in the brewing industry who often use the term. However, it is clear that bitterness perception is multifaceted” (McLaughlin et al., 2008; Oladokun et al., 2016a). “Furthermore, it is clear that some of bitterness attributes are in normal usage considered positive (‘harmonious’) whilst others (e.g. ‘harsh’) might be considered less desirable. In this regard, the intensity of bitterness corresponds to the magnitude of bitter taste sensation perceived, whilst temporal profile represents the time-course of bitterness intensity over a period of time” (Keast and Breslin, 2003). Sabo et al. (2001) deal with the content of active components depending on the number of lupulin glands in the hop cones.

“However, there is no scientific study on the impact of hop variety in relation to perceived bitterness quality in beer” (Oladokun et al., 2017). Slavík and Zavadil (2001), on the other hand, deal in their study with the need for supplemental irrigation in the Czech Republic.

In the first Czechoslovak Republic, hundreds of enterprises cultivated hops. In 1945, immediately after the war, the Druzstvo pestitelu chmele (Cooperative of Hop Growers) was founded, which in the following two years secured purchases and sales for their members. However, the cooperative's activities came to a halt after the Communist Party of Czechoslovakia came to power in February 1948, and the cooperative was nationalized. On New Year's Day 1960, two companies, Vykupni sklad chmele and Stanice pro pestovani chmele, merged to create the new national enterprise Chmelarstvi. After the Velvet Revolution in November 1989, the company was privatized and many hop-fields were returned in restitutions to their original owners. On 1 October 1992, the cooperative of hop-growers was given its current form, scope and name, Chmelarstvi, druzstvo Zatec. Nowadays, the trade in hops is the focus of the cooperative's subsidiary, Bohemia Hop (Chmelarstvi, druzstvo Zatec, 2017).

The purpose of this article is to describe the current state of the Czech and global hop market, hop production, and the extent of hop-fields, as well as the position of the dominant company, Chmelarstvi, druzstvo Zatec, on the market. Further, the article analyses the development of hop production and major related social and economic issues. Special attention will be paid to the increase in demand for Czech hops abroad in connection with the growing popularity of beers from microbreweries in the Czech Republic and the USA.

## 2 Materials and methods

The prevalent method used for the elaboration of this essay was the descriptive method (used for hop-growing in the Czech Republic). Furthermore, the method of comparative analysis was used to compare the outputs of individual hop-growing regions, while the statistical method was used to predict the further development of the size of hop-growing areas and hop production in the Czech Republic.

The specific research procedure will be as follows:

To look up data on global hop production in the statistics of the International Hop Growers' Convention (IHGC) and Food and Agricultural Organization (FAO) to clarify the issue of the market in hops, include the calculation of the Czech Republic's share in global hop production. Present the data in the form of a clear table.

To find the development of hop harvesting areas in the Czech Republic in the statistics of the Czech Statistical Office (CZSO). Subsequently, conduct a statistical analysis of the data by looking for the best (optimum) functions for estimating the development of hop harvesting areas and determine the function type according to the determination index. Graphically document the development of hop harvesting areas in the Czech Republic in the period from the country's accession to the EU to the present day and explain the development of the indicator.

To describe in the form of a graph the fluctuations in hop production in the examined period 2004–2017, as hop production depends not only on the current hop harvesting area, but also especially on the weather in any given year. Subsequently, study this development using statistical data analysis.

To express the distribution of hop production by individual areas, as hops are grown in several different locations.

To analyse the volume of production according to individual growers and determine whether there is a dominant hop producer on the domestic market.

Estimation of hop production in the coming years will be based on a simple prediction using the average growth coefficient.

The outcomes and recommendations for hop growers resulting from the analysis will be summarized in the final part of the study.

In order to verify the current position of Czech hop production on the international market and in the Czech economy, the following hypothesis was established: *"Czech hop production is a promising sector of the domestic economy due to the long tradition of hop growing in the Czech Republic, the increasing area of hop fields, and also the increasing demand for Czech hops from breweries in the Czech Republic and abroad."*

### 2.1 Analytical smoothing of time series

The shortage of graphical and mechanical time series smoothing can be corrected using analytical methods based on expression of the time series track by the mathematical function (see Equation (1)).

(1) $$y_{t}^{\prime }=f(t)+e_{t}\;,$$

$y_{t}^{\prime }$ is the theoretical (smoothed) value of the time series indicator studied, *t* is the time variable, i.e., ordinal numbers of the time series sequence *(t = 1, 2, …, n)*, *f(t)* is the *t* time variable function, *e*_*t*_ is the residual component.

The function parameters can be obtained using the least squares method (see Equation (2)).

(2) $$\sum_{t=1}^{n}{\left(y_{t}-y_{t}^{\prime }\right)}^{\;2}=\min,$$

*y*_*t*_ is the empirical (observed) time series values *(t = 1, 2, …, n)*, $y_{t}^{\prime }$ is the theoretical (expected) value, obtained by the trend function *(t = 1, 2, …, n)*.

The basis of the decision on the appropriate trend function type was the factual economic criteria (Taylor, 2007). Finding the right type of trend function is then supported firstly by the analysis of empirical data. The criterion applied is the index of determination (see Equation (3)).

(3) $$I^{2}=1-\frac{\sum_{t=1}^{n}{\left(y_{t}-y_{t}^{\prime }\right)}^{2}}{\sum_{t=1}^{n}{\left(y_{t}-\bar{y}\right)}^{2}}.$$

Some further criteria for the choice of an appropriate model have been applied in the paper. These are based on the comparison of the sum of the squares of deviations between the empirical and theoretical values. It is important, anyway, to realize that none of these criteria have a universal character; they offer partial information only on the quality of the model studied (Hindls et al., 2007; Freedman et al., 2007). For significance testing of the models and their parameters, the α = 0.05 significance level has been chosen. Statistical computations have been done in the STATISTICA software, version 13.

### 2.2 Global hop production and its regional aspects

In July 2016, the International Hop Growers' Convention (IHGC) (2018) Economic Commission estimated the global area of fields on which hops are harvested at 54,614 ha. The greatest volume of aroma hops (including the flavour hops varieties) is grown in the US. Globally, the area on which aroma hops are produced makes up almost 70% of the total area of hop fields. Five years ago, it was only half. The leading global position of the US in hop production, as well as the position of the Czech Republic as the third largest hop producer in the world, are evident from Table 1 below.

**Table 1:** Global hop production, producers with a harvest of 800 tonnes and over in 2016.

Rank	Country	Hops Production (in tonnes)
1	United States	39,526
2	Germany	32,053
3	China	8,489
4	Czech Republic	7,712
5	Slovenia	2,478
6	North Korea	2,030
7	Poland	2,003
8	Albania	1,967
9	Spain	967
10	New Zealand	809
	Total global production	104,110

In the ***Central European Region***, hops are grown mainly in Germany, the Czech Republic and Poland. The Federal Republic of Germany, where 30.79% of the world's hop crop was produced in 2016, holds the dominant position in this area.

**Figure 1:**
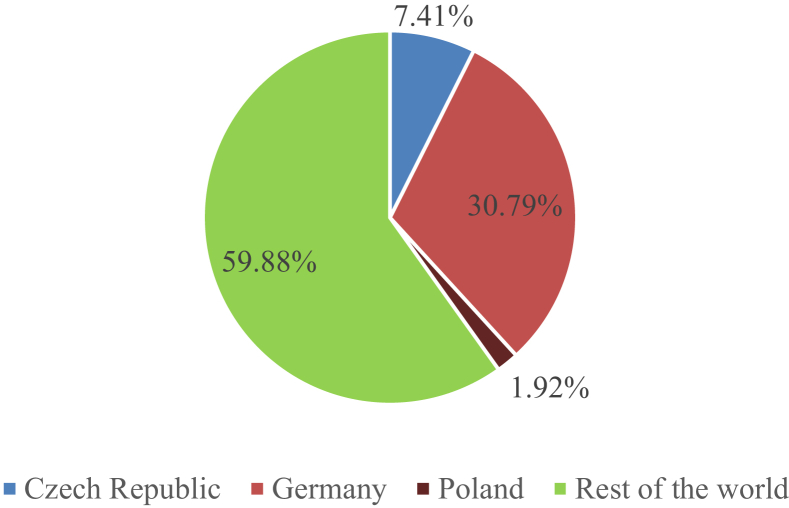
Comparison of hop production in the Czech Republic, Germany and Poland with global production in 2016. *Source*: Faostat (2018).

Another large producer of hops in Central Europe is the Czech Republic, which is the third largest hop grower in the world and the second largest hop grower in Europe, with its 7.41 % share of global hop yields. Poland holds third position among Central European hop growers with its almost 2% share of global hop yields, which shows the success of hop cultivation in this region. However, as shown in Figure 1, Poland's share of hop production in Central Europe is incomparably smaller than that of its neighbouring countries, i.e. Germany and the Czech Republic. We can thus conclude that hop cultivation in Central European countries is a success and that the region's share of more than 40% of global hop yields proves its global importance in the production of this commodity.

### 2.3 Historical trends in hop cultivation in the Czech Republic

Since the time of the first Czechoslovak Republic, the country has been among the hop producing superpowers. With an almost one-tenth share of the harvest, the Czech Republic is the third largest producer of aroma hops in the world after Germany and the US, which together account for over two-thirds of the hop market.

**Figure 2:**
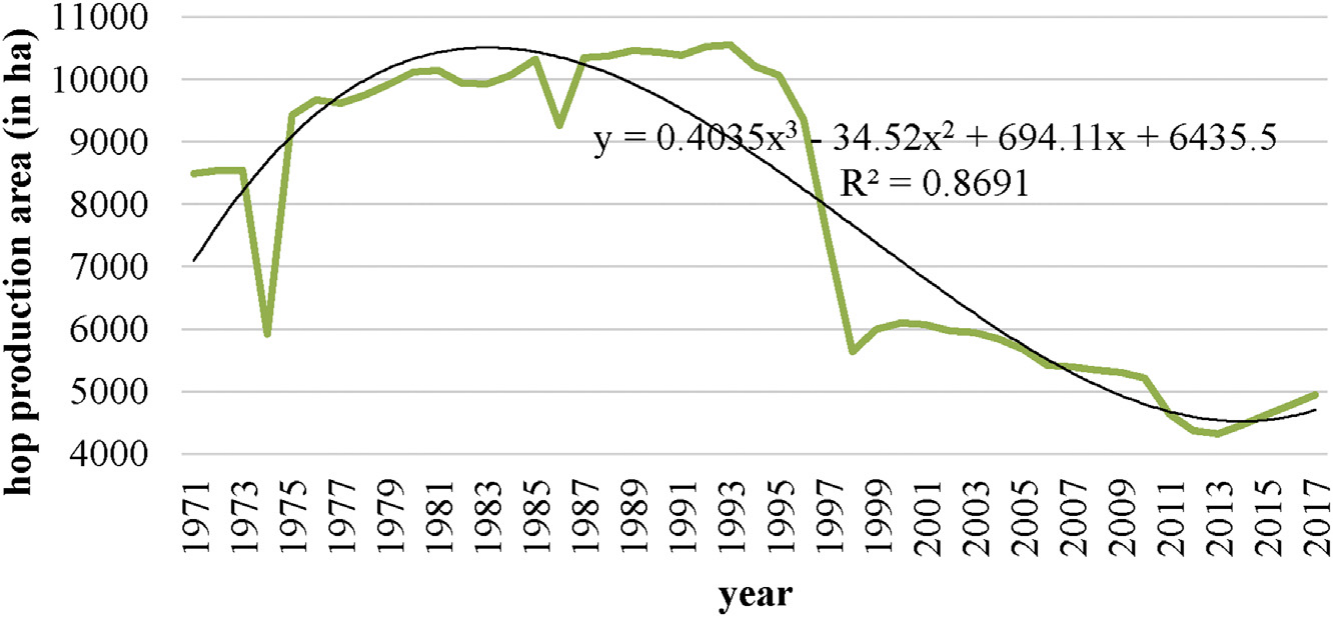
Long-term development of hop production areas in the Czech Republic from 1971 to 2017 (in ha). *Source*: CZSO (2018).

Figure 2 shows the long-term trends in hop-growing areas of the Czech Republic from 1971 to 2017, including a description of the trends with a third degree polynomial function.

The development of agriculture after 1989 has significantly been influenced by the change in the structure of ownership relations of agricultural land (restitution, private enterprises) and by an increased pressure to rationalize work (Beranová et al., 2017). In the early 1990s, hops were grown on over 10 thousand hectares in the Czech Republic. With the advent of a market economy came a decline, which stopped only in 2013 at 4,319 ha. Paradoxically, growth in the area of hop-fields was caused by the bad harvests of 2012, 2013 and 2015.

Due to the bumper harvest of 2010, when not all the crops could be sold, some growers ended hop production. The reasons for the decrease in the area of hop fields in recent years have also been economic, as Czech hops, mostly of the Saaz variety, are globally considered to be among the highest quality varieties, and therefore also among the most expensive. This economic reason is why some beer brewers switched to cheaper varieties of hops.

Among other economic reasons was also a drop in demand from one of the largest markets for domestic hops, Japan, whither hops have been exported since 1905. The fact that the world's largest brewery, Anheuser Busch, then terminated its contracts with the Žatec cooperative due to trademark conflicts with Budweiser Budvar Brewery also had its effect. A significant role was played by the pressure on breweries to reduce costs, which meant a drop in demand for hops.

### 2.4 Area of hop fields in the Czech Republic and its development

Most of the original hop fields are gone irretrievably. On the majority of former hop-fields, support structures were removed without which hops cannot be grown, and other crops are being grown there. Many original hop fields were only rented by the grower, and if the land owner does not agree with the restoration of hop-growing, the potential hop grower will not succeed.

**Figure 3:**
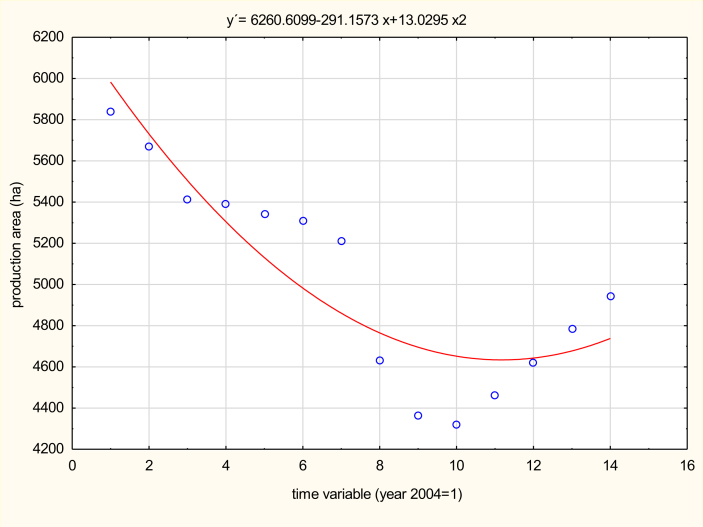
Area of hop fields in the Czech Republic after the country's entrance to EU in the period 2004–2017 (in ha). *Source:*CZSO (2018)*; own processing*.

Nevertheless, it seems that the sector has already reached its bottom, and for the fourth year now the area of hop fields has grown slightly. In 2014, the area of hop fields grew for the first time since 2000 (Figure 3). It reached 4,460 ha, which is 3% more year-over-year. Hop field area has been decreasing for almost 20 years; in 1995, hops were grown on over 10,000 ha.

Towards the end of 2016, the area of hop fields in the Czech Republic reached 4,783 ha, which is the most in the last 6 years. Year-over-year, this represents an increase of 3.5%. The increase was caused by the current demand for quality Czech hops and the weak European harvest in 2015 (Svaz pěstitelů chmele, 2018). Thus the storehouses emptied and breweries' stores all over the world, to which 80% of the domestic hop production is supplied, ran out. Since then, demand and prices have increased to a level that has begun to cover the costs of hop production. This has also meant that investment in hop production has increased.

It is also encouraging that since 2014, thanks to support from the Ministry of Agriculture, growers have embarked on the extensive restoration of hop-fields. In 2014 alone, 406 new hop fields were established, which is the double the number from the previous years. Market interest in fine aroma hops remains, so growers are not losing their optimism and are still planting new hop fields. It also shows the further expansion of the hop-growing area in the Czech Republic in 2017 to 4,945 ha, i.e. a year-on-year increase of 3.39%. The target of hop growers is to overcome the five-thousand-hectare limit within the next few years.

However, Czech hop production faces two fundamental problems. The first of them is the age of the domestic hop fields, and the second is their obsolete supporting structures. The average age of these structures is increasing and is even less favourable than the age of hop plants, as 65% of the structures are over 20 years old. This might change, as in the new EU programming period, hop-growers are given special support and hops are included among the sensitive commodities. Besides, the increase in the number of new hop fields is positive, as well as the rate of renovation of the old ones. In 2015, 7% of existing hop fields were renovated.

### 2.5 Hop production in the Czech Republic

**Figure 4:**
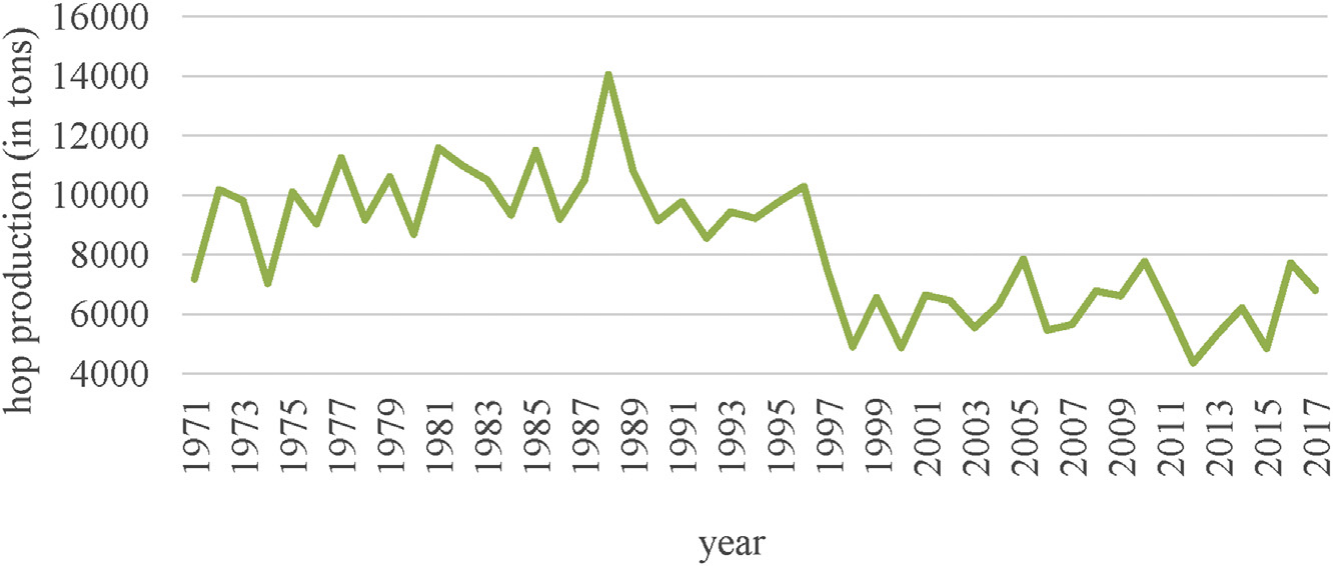
Long-term development of hop production in the Czech Republic from 1971 to 2017 (in tons). *Source*: CZSO (2018); *own processing*.

In the Czech Republic, hop production greatly depends on the weather in the given year in the regions where hops are grown. Figure 4 shows the development of hop production in the Czech Republic from 1971 to 2017. In 2015, therefore, due to droughts, the hop harvest reached only 4,843 tons, with a low yield of 1.05 tons per hectare.

“The hop cannot be cultivated in the Žatec region profitably without modern economical irrigation” (Slavík, 2000). “In the conditions of Krušné hory rain shadow hop plants irrigation has a great importance for economic effectivity of hop growing. The shortage of water negatively influences the course of physiological processes, such as the growth and photosynthesis. Accumulation of energy matters is affected by this and finally also the yield formation. Accumulation of energy in hop plant is of a dynamic character in the course of vegetation” (Hniličková and Novák, 2000).

The second factor significantly influencing total hop production is the area of hop fields on which hops are grown in the given year.

**Figure 5:**
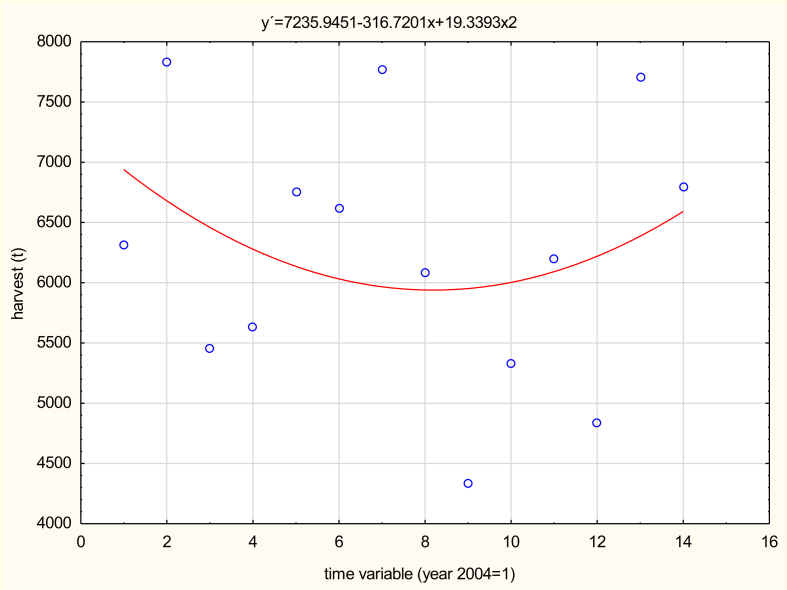
Variability in hop production in the Czech Republic in the period 2004–2017 (in tons). *Source:*CZSO (2018)*; own processing*.

In 2016, a total of 7,711.61 tons of dry hops were harvested in the Czech Republic (Figure 5), with the average yield per hectare being 1.61 tons, historically one of the best results. Compared to 2015, an extra 2,868.99 tons of hops were harvested, which represents an increase of 59.24% (Central Agricultural Inspection and Testing Institute, 2016). Increased precipitation towards the end of the vegetation season was used primarily by the later hybrid varieties, causing a significant increase in the yield. In 2016, although the crop harvest was unexpectedly large, due to the accumulated shortage of hops from previous smaller harvests, there is no doubt that the entire hop harvest will sell well.

In 2017, the amount of hops harvested in the Czech Republic was 6,797 tons, which is 915 tons less than in the previous year, 2016, and represents an 11.86% decrease in hop production. This worse result in hop production was caused by weather fluctuation, both the winter and spring frosts and the dry summer, in particular.

### 2.6 Hop yields in the Czech Republic

The indicator hop yield per hectare is measured as the ratio between the hop production in the given territory and the hop-growing area in the same period - usually a one-year period. “Agricultural yield is the key determinant of cost variations” (Maitah et al., 2019).

**Figure 6:**
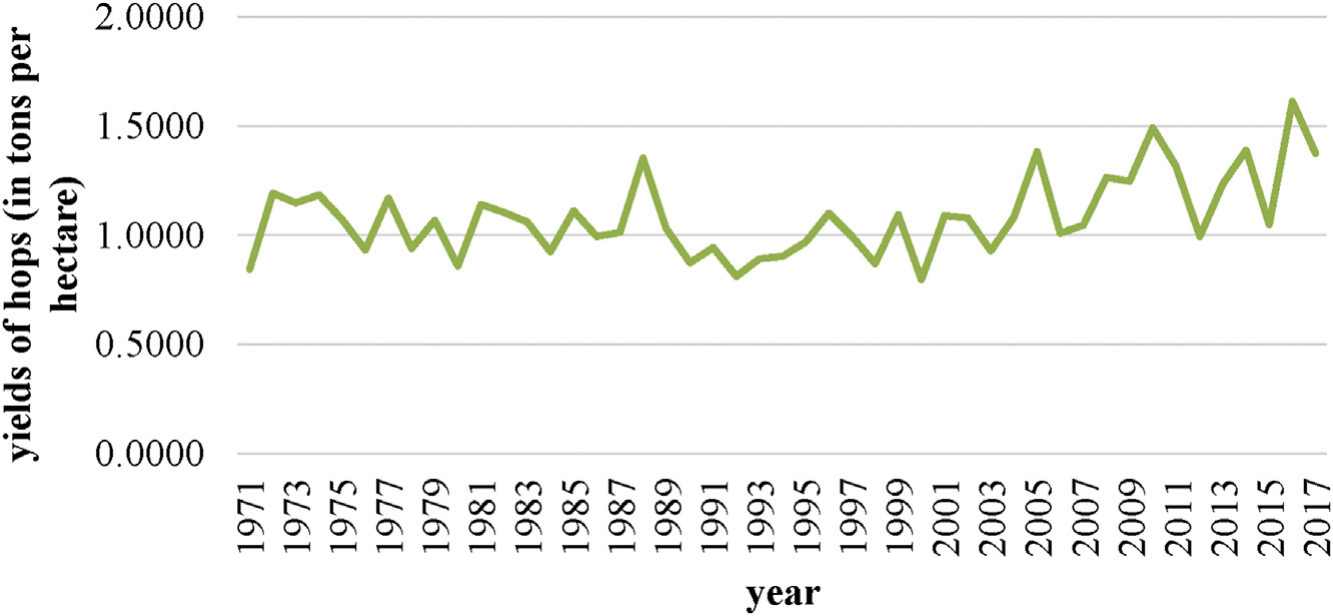
Long-term hop yield trends in the Czech Republic for the period 1971–2017 (in t/ha). *Source*: CZSO (2018); *own processing*.

The hop yield indicator in t/ha is thus influenced by both the change in hop production (e.g. as a result of climatic or weather changes) and by the area of the hop yards in the region. For example, in 2017 there was a 3.39% increase in the hob-growing areas compared to 2016, but the effect of the 11.86% decrease in production caused by frosts and droughts led to an overall decrease of 14.75% in the hop yields per hectare of the hob-growing area (see Figure 6).

**Figure 7:**
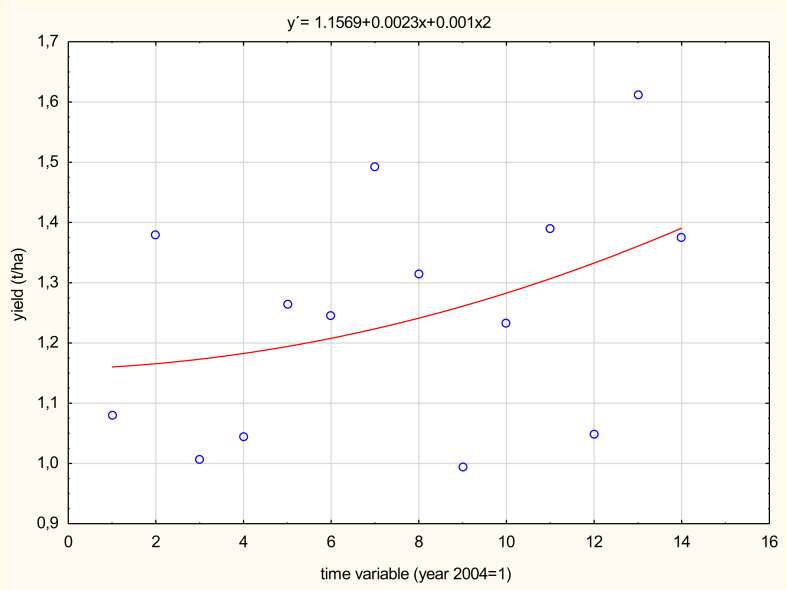
Development of hop yields in the Czech Republic for the period 2004–2017 (in t/ha). *Source:*CZSO (2018)*; own processing*.

The average hop yield per hectare in the Czech Republic shows considerable variability in 2004–2017 (see Figure 7). Nevertheless, a slight increase in yields can be seen in recent years due to the breeding work of specialist workplaces. The increase in hops yields in the Czech Republic in recent years is thus undoubtedly due to the cultivation of hop varieties with higher yields, but also due to the selection of suitable soil during the establishment of new hopfield.

In particular, the end of the growing season is important for the maturation of hop plants. According to the latest data, precipitation was above the long-standing average in June and July 2016. Alternating rain and warm weather conditions increase the infection pressure of diseases. However, hop growers are making every effort to prevent diseases and are able to maintain the stands in a healthy condition despite the increased costs.

Nevertheless, the infection pressure of Pseudoperonospora humuli was very high in this period. Due to frequent chemical treatment, the growers managed to maintain stands on most of the growing areas in good health. On the other hand, 130–150 ha of hops were almost completely destroyed by hailstorms at the turn of May/June 2016. At the turn of July/August 2016, hail fell on a total of 20 ha of hop fields in storms accompanied by strong winds in Žatec and Moravia. Dozens of other hop bushes fell and had to be hung up again (Central Agricultural Inspection and Testing Institute, 2016).

### 2.7 Distribution of hop production in the Czech Republic by hop-growing area

**Figure 8:**
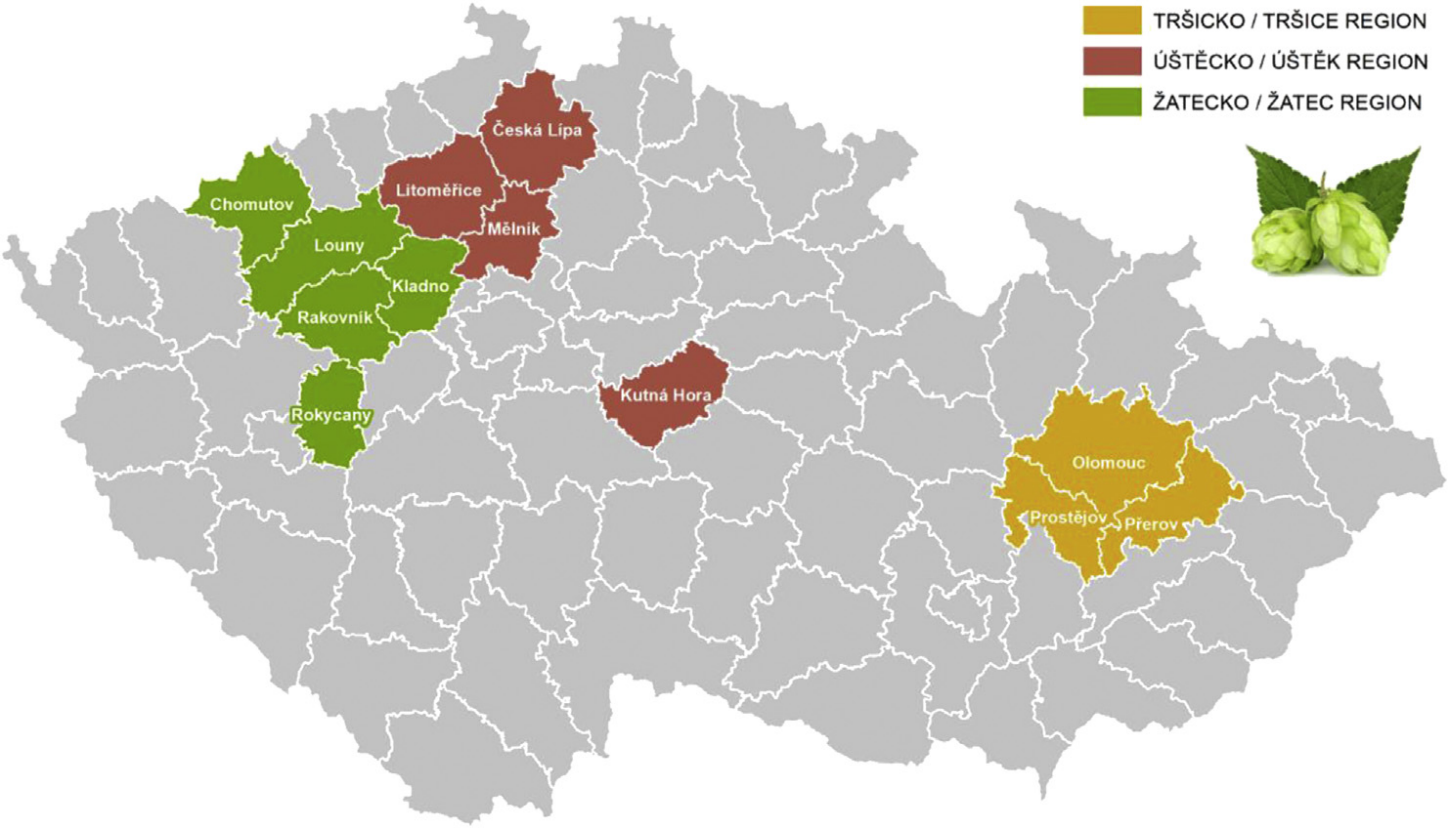
The map of hop-growing areas in the Czech Republic. *Source: Ministry of Agriculture of the Czech Republic, 2020; own processing*.

A typical hop-growing area in the Czech Republic, and also the largest, is the Žatec area (which includes the districts of Louny, Rakovník, Kladno, and Chomutov, as well as the districts of Plzeň-North and Rokycany, which no longer actively cultivate hops), where most of the Czech hopfields are located. Another important hop-growing area is Úštěk and Haná in Moravia, in particular the area surrounding Tršice (see Figure 8).

Unlike 2015, 2016 was very favourable for the production of hops. It was so for the largest hop producer in the Czech Republic, Chmelarstvi, druzstvo Zatec (see Figure 7). In the Žatec Region in 2016, hop production increased to 5,799.77 tons, which is 2,331.18 tons more than in 2015, the increase being 67.21%. Compared to 2015, the production of the Saaz variety increased by 2,060.03 tons, to 4,953.91 tons, which is an increase of 58.75% (Central Agricultural Inspection and Testing Institute, 2016).

In 2016, the Tršice area (in the Olomouc Region) saw a year-over-year increase in hop production of 40.54%. 1,092.53 tons of hops were harvested. Compared to 2015, this was an extra 315.17 tons. An extra 235.11 tons of the Saaz variety were harvested (749.35 tons total), which is an increase of 45.72% (Central Agricultural Inspection and Testing Institute, 2016).

From among the hop-growing regions, the Úštěk area (the Ústí Region) showed the lowest increase in harvested hops as a percentage: merely 37.31%. 819.31 tons of hops were harvested, i.e. 222.64 tons of hops more than in the previous year, 2015. An extra 167.96 tons of the Saaz variety were harvested, which brings the total to 663.25 tons and represents a year-over-year increase of 33.91% (Central Agricultural Inspection and Testing Institute, 2016).

**Figure 9:**
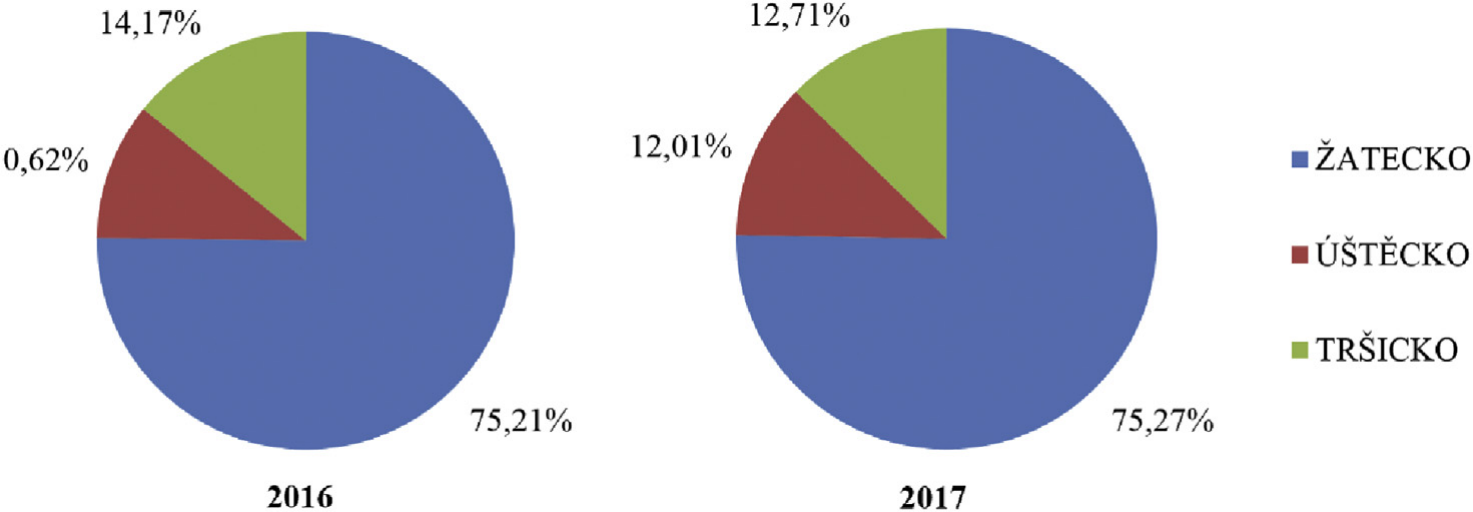
Changes in the distribution of hop production in the Czech Republic by hop-growing area between 2016 and 2017 (in %). *Source: Central Agricultural Inspection and Testing Institute, 2018; own processing*.

The differences in hop production in the Czech Republic between 2016 and 2017 in individual hop-growing regions are evident from Figure 9. The increase in the share of the Úštěk area, and conversely the decrease in the share of the Tršice area, in nationwide hop yields is undoubtedly related to climatic changes, which currently affect the yields of agricultural crops in much of Europe. For the second year in a row, the South Moravian Region has suffered from a lack of water in the soil due to decreased precipitation along with higher summer temperatures. The second factor influencing the shares of hop production among regions is changes in the area of hopfields, for example the increase in the area of hopfields in the Úštěk area.

**Figure 10:**
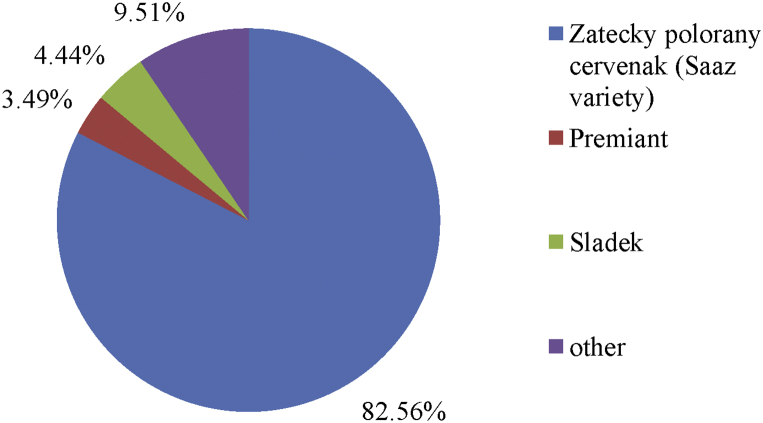
Distribution of hop production in the Czech Republic by hop variety in 2016 (in %). *Source: Central Agricultural Inspection and Testing Institute 2017; own processing*.

The achieved hop yields per hectare are influenced not only by the climatic and weather conditions of a particular year, but also by the mix of hop varieties (see Figure 10). “Zatecky polorany cervenak” is the dominant hop variety in the Czech Republic, making up 82.56% of the total hop production in 2016. The Sladek variety was in second place with a 4.44% share, before the Premiant variety in third place with 3.49%. The remaining 9.51% falls to the other varieties grown in the Czech Republic.

### 2.8 Chmelarstvi, druzstvo Zatec – dominant company in the field of hop production

The fact that the Czech Republic is currently the third largest global hop producer, after Germany and the US, is to the credit of the hundreds of hop growers associated in Chmelarstvi, druzstvo Zatec, which manages 4,265 ha of hop fields in the Czech Republic. The area of hop fields cultivated by the cooperative Chmelarstvi, druzstvo Zatec corresponds to approximately 95% of the entire area of hop fields in the Czech Republic, and it has been so throughout the entire history of the cooperative, whose roots go back to the period immediately after World War II. This was the case even in the 1990s, when the cooperative had double the number of members it has at present (Chmelarstvi, druzstvo Zatec, 2017).

To determine the dominance of a company on the domestic market, the Office for the Protection of Competition in the Czech Republic uses the criterion of the company having a 40% share in the given market. In the case of hops, the hop production area and the hop production create an aggregate indicator of a company's dominant position on a relevant market.

## 3 Results

### 3.1 Investment in expanding area of hop fields

The goal of hop growers in the Czech Republic is to pass once again the five-thousand-hectare limit to the area of hop fields. The future increase in the area of hop fields will depend primarily on the opportunity to buy land for hop field preparation and planting, and on the willingness of land owners to lease out land to hop growing companies for at least ten to fifteen years. “The role of agricultural credit on farmers' welfare needs to be investigated” (Essossinam and Essossolim, 2019).

### 3.2 Prediction of the hop-growing area, production and yields

The exploratory analysis of the time series of the hop-growing area, production and yields (Table 2) indicates a higher variability for production and yields for the reasons stated above. Therefore, a prediction of the hop-growing areas only was made in the next part.

**Table 2:** Description of yield-increasing characteristics of hop varieties in the Czech Republic in 2001–2017.

Variable	Descriptive statistics					
	Valid N	Mean	Minimum	Maximum	Std. Dev.	Coef. Var.
Production area (ha)	14	5,021.571	4,319.000	5,838.000	497.157	9.90042
Harvest (t)	14	6,262.643	4,338,000	7,831.000	1,080.237	17.24890
Yield (t/ha)	14	1.249	0.994	1.612	0.193	15.43292

#### 3.2.1 Prediction of the development of hop production areas in the coming years

Based on the analysis of the data about the development of the hop production area in the Czech Republic between 2004 and 2017, a model was selected and put together for the prediction of parameters for the 2018 to 2020 period. The highest value of the determinant showed a second degree polynomial (Table 3).

**Table 3:** Model Summary and Parameter Estimates (own work).

N = 14	Regression Summary for Dependent Variable: production area (ha) R = .89582321 R^2^ = .80249923 Adjusted R^2^ = .76658999 F (2,11) = 22.348 p < .00013 Std. Error of estimate: 240.19					
	b∗	Std. Err. of b∗	b	Std. Err. of b	t (11)	p-value
Intercept			6,260.610	223.7927	27.97504	0.000000
time variable	-2.44993	0.577549	-291.157	68.6378	-4.24194	0.001384
time variable∗∗2	1.69068	0.577549	13.030	4.4510	2.92733	0.013756

Point forecasts and 95% interval forecasts of hop production areas for 2018–2020 are given in Table 4.

**Table 4:** Point and 95% interval forecasts of hop production areas in the Czech Republic for 2018–2020 (in ha).

Year	Point forecast	Lower limit of interval	Upper limit of interval
2018	4,824.90	4,332.33	5,317.46
2019	4,937.65	4,297.58	5,577.72
2020	5,076.47	4,266.30	5,886.64

According to the model selected (Table 4), the hop production area in the Czech Republic will increase at a moderate pace, and in 2020 it should reach 5,076.47 ha. This would raise the hop production area to the level it was at in 2010.

However, investing in a new hop field is not cheap. According to rough calculations, establishing a hop field of 50 ha, including related technologies, costs approximately CZK 100 million (EUR 3.7 million). One third of the cost goes to the construction of hop growing structures. Harvesting machines, a drying hall and a storage hall cost another third and the rest is for equipment such as sprayers, bine removers, cultivation machines and others (Kütner, 2016b).

The return for other common investments is expected within five to seven years. For hops, the growers have always anticipated returns on investments within 15 years. This period has shortened slightly with increasing interest in the expansion of hop fields and domestic hops. The state subsidy policy will also make a difference. “There have recently been a number of changes to the Common Agricultural Policy (CAP), mainly as a result of the addition of the new Member States from Eastern Europe” (Morley and Morgan, 2008). “For example, in preparation for European Union (EU) membership and adoption of the Common Agricultural Policy (CAP), most acceding states introduced direct payments for farmers and other measures that mirrored those in use in the EU” (Chaplin et al., 2007). “The necessity for agriculture to receive state support, including financial support, aimed at stimulating growth in its efficiency, is determined by the characteristics of the agrarian sector” (Maitah et al., 2016). The Ministry of Agriculture intends to support the planting of new hop fields, including the matter in its new strategy with the outlook to 2030.

Due to the adjustment in subsidies for sensitive commodities by 2020, the Ministry of Agriculture of the Czech Republic intends to maintain the area of hop fields at around five thousand hectares, and after 2020 to increase this to approximately 5,500 ha. There are several subsidy programmes to support hop growing, within which the state paid out over CZK 16 million (EUR 592 thousand) in 2015. Within the support for sensitive commodities, among which hops were included few years ago, the state paid out further subsidies of over CZK 17 thousand (EUR 629) per hectare (Ministry of Agriculture of the Czech Republic, 2016).

## 4 Discussion

### 4.1 Increase in the number of hop varieties in the CR

Hop cultivators are meeting the trends in hop-growing and beer-brewing, including the emergence of microbreweries and the special kinds of beer they concentrate on. The Czech Republic, as a significant global hop producer, is facing the problem of the emergence of new varieties. The number of hop varieties might double (currently, twelve varieties are grown in domestic hop fields). In the majority of planted hop fields, the traditional and globally sought-after Saaz variety is grown. In the coming years, the list should expand by a further eleven varieties.

These are not to replace the existing quality varieties, but to expand the variety of substances that are valuable in beer brewing. Hop cultivation, which is one of the major tasks of Hop Research Institute Co., Ltd., Saaz, has lately been going in four major directions: aroma varieties, bitter varieties, low-trellis varieties and the special aroma hop varieties used in particular types of beer, like ale, IPA and wheat beer (Kütner, 2016b).

Aroma varieties remain in use for Czech beers, as the consumers know them. Bitter varieties and those with a specific aroma will expand the variety for both industrial breweries and, in particular, small-scale breweries that keep bringing out new brands. The introduction of the first low-trellis varieties will be somewhat of a novelty. Many hop growers have been experimenting with this new technology, but so far there has been no suitable Czech variety. The names of new hop varieties are not known yet, but they might be named after the planets (Saturn, Jupiter, Mars, etc.).

### 4.2 Increase in demand for Czech hops from microbreweries

New market opportunities are opening to domestic hop growers. Not only the Czech Republic, but North America, too, has seen an increase in the popularity of microbreweries. This results in a much-increased interest in Czech hops, which Czech producers have not been able to meet fully in recent years due to bad harvests. Therefore, new deals on the coming harvests will be possible only if the harvest is above-average.

The higher estimate for harvests in the subsequent period (Table 4) might saturate the increased demand for Czech hops from abroad. Increased interest in Czech hops was confirmed at the traditional Craft Brewers Conference in Philadelphia, US in 2016. There were many craft breweries from the US and Canada (including the largest TOP 10 to TOP 20 craft breweries), newly interested in Czech hops.

In 1970 the number of microbreweries in the United States was around one hundred. A sharp increase came at the end of the last decade, and in 2015 their number increased to 4,225 (Table 5). At present, there are over 4,700 microbreweries in the United States. Almost three fifth are microbreweries with annual production of up to 18 thousand hectolitres; just under two fifths are restaurant breweries with even lower production. Four percent are craft breweries with annual production between 18 thousand and seven million hectolitres (Brewers Association, 2016).

**Table 5:** Development in the number of microbreweries in the US.

	2012	2013	2014	2015
Craft breweries	97	119	135	178
Microbreweries	1149	1464	2041	2397
Restaurant breweries	1155	1280	1500	1650
Total	2401	2863	3676	4225

However, the total volume of Czech hops produced has practically sold out for the next few years. According to the Bohemia Hop company, the contracted quantities will increase if the harvest is good. It is estimated that the absorption of the American market would allow an increase in the export of Czech hops of one hundred percent, or even more, within three years (Kütner, 2016a). US craft breweries prefer the top fermenting ales and India pale ales. This is why many hop growers abroad, including the largest competitors in Germany, have begun to grow hops suitable for these types of beer and limited their production of hops suitable for bottom fermenter lagers, like the Saaz variety. This has contributed to the fact that Chmelařství, družstvo Žatec has seen a greater demand for Czech hops abroad for lager beers.

The countries to which Czech hops are exported have somewhat changed in recent years. Japan, where almost two fifths of Czech production used to go, have lowered their demand, with China making up for this. The most populated country in the world is experiencing a boom in beer brewing, and currently one fourth of global production comes from its breweries.

### 4.3 System of determining the origin of Czech hops

There is such great demand for Czech hops that from time to time attempts to sell hops of lesser quality under the name Saaz occur. To deal with this issue, the Czech Republic has developed a certification system and a system for determining the origin of hops. This is why, currently, the journey of hops can be traced back from a brewery to a particular hop field. *Chmelarstvi, druzstvo Zatec* owns analytic tools that can determine how much of the Saaz variety there is in a sample, if any at all. Customers often send samples of hops to Žatec for verification, to make sure that they have bought genuine Saaz (Chmelarstvi, druzstvo Zatec, 2017).

### 4.4 Shortage of workers in hop growing

With the increasing area of hop fields in the Czech Republic, the hop growers have to deal with a significant problem, which is the unavailability of workers. Hop growing includes two seasons, when for a few weeks an extra several thousand temporary workers are needed - in the spring, when the cables need to be stretched on trellises for the hops to climb, and in the autumn, at the harvest. In the past, secondary schools and universities all over the country greatly helped the growers, sending whole grades to work in the hop fields. However, this stopped in the 1990s, and the share of secondary schools in the spring work is minimal. Although individual students come for the harvest, these are mostly people from the area or students sent by work agencies. Agency workers are prevalent among temporary workers as they send mostly adult workers, not only from the Czech Republic, but also, for example, from Slovakia, Romania and Bulgaria.

Nowadays, seasonal work in hop fields differs greatly from what we knew twenty and more years ago. The tasks of seasonal workers during the hop harvest, especially, are different now, as it no longer involves manual harvesting. Nowadays, the process of harvesting is mechanized, using adapted tractors. Seasonal workers collect the remnants of unharvested hops, clean the hop fields, dry, bag and tidy.

Unlike their parents, the seasonal workers at the hop harvest do not work for free. On the contrary, this is quite a well-paid job. In addition to the basic hourly rate, the workers receive performance bonuses and bonuses for meeting the set harvest targets. This is reflected in the interest in this kind of temporary work.

As the demand for hops increases, so does the demand for temporary workers to help harvest them. There are two possible solutions to the labour shortage:

• An increase in the number of foreign workers (quotas) that are seasonally employed in the hop-growing sector (in particular from Ukraine, where unemployment is high) can improve the standard of living of their families in their home country. There are many studies which discuss the subject of social & economic relations as a result of the immigrant's access to accommodation and the labour market (Nová, 2016, 2018).
• The introduction of Industry 4.0 knowledge in hop production and harvesting. Since most of the special technologies are produced by Chmelařství Žatec, it will depend on their willingness to put this knowledge into practice and thus replace the currently missing workers.

### 4.5 Evaluation of the analysis and the validity of the hypothesis

Based on the analysis of specific data on the growing and harvesting of hops in the Czech Republic, the prospects for hop production as an important sector of the Czech economy can be confirmed. Beers produced in the Czech Republic using local varieties of hops, the Saaz variety in particular, have become popular with consumers, whether domestic or foreign. The boom of microbreweries and the growing popularity of beer worldwide contribute to sales of Czech hops on international markets. The high quality of products of the Czech beer industry which stems from the quality of Czech hops, is generally known and provides jobs for many people in the hospitality industry in the Czech Republic and abroad.

Therefore, we can confirm in principle that the validity of the given hypothesis on the prosperity of the Czech hop growing sector has been confirmed with the obtained research results. On the other hand, it must not be forgotten that there are certain limits to the development of the hop industry in the Czech Republic, both in the area of investment in creating new hop fields and in providing seasonal workers for work in the hop fields. Presently, the unemployment rate has dropped to less than 3% and there is a lack of workers in the majority of sectors of the Czech economy, and therefore also in hop fields.

### 4.6 Resulting recommendations

For the successful future development of the Czech hop production sector, the following ***recommendations*** resulting from the prepared study can be proposed:

Market interest in fine aroma hops remains. The goal of hop growers in the Czech Republic is to pass once again the five-thousand-hectare limit to the area of hop fields. The future increase in the area of hop fields will depend primarily on the opportunity to buy land for hop field preparation and planting, and on the willingness of land owners to lease out land to hop growing companies for at least ten to fifteen years.

The average age of these structures is increasing and is even less favourable than the age of hop plants, as 65% of the structures are over 20 years old. This might change, as in the new EU programming period, hop-growers are given special support and hops are included among the sensitive commodities.

In the Czech Republic, hop production greatly depends on the weather in the given year in the regions where hops are grown.

New market opportunities are opening to domestic hop growers. Not only the Czech Republic, but North America, too, has seen an increase in the popularity of microbreweries. This results in a much-increased interest in Czech hops, which Czech producers have not been able to meet fully in recent years due to bad harvests. It is estimated that the absorption of the American market would allow an increase in the export of Czech hops of 100%, or even more, within three years.

Hop cultivators are meeting the trends in hop-growing and beer-brewing, including the emergence of microbreweries and the special kinds of beer they concentrate on. The number of hop varieties might double (currently, twelve varieties are grown in domestic hop fields). These are not to replace the existing quality varieties, but to expand the variety of substances that are valuable in beer brewing.

## 5 Conclusion

Since the time of the first Czechoslovak Republic, the country has been among the hop production superpowers. Towards the end of 2017, the area of hop fields in the Czech Republic reached 4,945 ha, which is the most in the last 7 years. Year-over-year, this represents an increase of 3.4%. The increase was caused by the current demand for quality Czech hops and the weak European harvest in 2015. Thus, the storehouses emptied, and breweries' stores all over the world, to which 80% of domestic hop production is supplied, ran out. Since then, demand and prices have increased to a level that has begun to cover the costs of hop production. This has also meant that investments in hop production have increased. With the increasing area of hop fields in the Czech Republic, hop growers have to deal with a significant problem, which is the unavailability of workers. The state subsidy policy will also make a difference. The Ministry of Agriculture intends to support the planting of new hop fields, including the matter in its new strategy with outlook to 2030.

## Declarations

### Author contribution statement

K. Šrédl: Conceived and designed the experiments; Contributed reagents, materials, analysis tools or data; Wrote the paper.

M. Prášilová: Conceived and designed the experiments; Analyzed and interpreted the data; Contributed reagents, materials, analysis tools or data; Wrote the paper.

R. Svoboda: Performed the experiments; Analyzed and interpreted the data; Contributed reagents, materials, analysis tools or data; Wrote the paper.

L. Severová: Analyzed and interpreted the data; Contributed reagents, materials, analysis tools or data.

### Funding statement

This work was supported by the Internal Grant Agency of Faculty of Economics and Management, Czech University of Life Sciences in Prague (2020B0002) – The impact of climate change on the economic performance of the viticulture and winemaking sector in the Czech Republic.

### Competing interest statement

The authors declare no conflict of interest.

### Additional information

No additional information is available for this paper.
